# The clinical value of P-wave terminal force in lead V1 in evaluating pericardial thickness in tuberculous constrictive pericarditis

**DOI:** 10.1186/s13019-024-02526-z

**Published:** 2024-02-12

**Authors:** Yanhong Ren

**Affiliations:** Affiliated Hangzhou Chest Hospital, Zhejiang University School of Medicine, Hangzhou, China

**Keywords:** P-wave terminal force in lead V1, Tuberculous constrictive pericarditis, Pericardial thickness

## Abstract

**Aim:**

To investigate the relationship between p wave terminal force (Ptfv1) and pericardial thickness in patients with tuberculous constrictive pericarditis.

**Methods:**

From January 2018 to October 2022, 95 patients with tuberculous constrictive pericarditis who needed pericarditis dissection in a hospital were collected, and 3 patients who did not meet the criteria were excluded, a total of 92 cases. The absolute value of Ptfv1 in conventional electrocardiogram was tested before surgery, and pericardial thickness was measured by echocardiography and chest CT. Pericardial thickness was measured after pericardial dissection. Pearson correlation analysis was used, R software was used to make scatter plot, and non-parametric square test was used. The correlation of postoperative measurements with echocardiography, chest CT and absolute value of Ptfv1 was analyzed.

**Results:**

Pearson correlation analysis was conducted with postoperative measurements and echocardiography measurements, postoperative measurements and chest CT measurements, and postoperative measurements and absolute value of Ptfv1. Pearson correlation analysis showed that the correlation coefficients between postoperative measurements and echocardiography, chest CT and Ptfv1 values were statistically significant. Scatter plot and nonparametric Chi-square test showed that postoperative measurements were consistent with absolute values of echocardiography, chest CT and Ptfv1 (*p* < 0.05). And this study found that the distribution of the value of Ptfv1 ≥ 5 was higher than the value of Ptfv1 < 5 after pericardiectomy (0.95:0.05) in the absolute value of Ptfv1 ≥ 0.04 which measured before pericardiectomy. The hypothesis was statistically significant (*p* < 0.05).

**Conclusion:**

The absolute value of Ptfv1 in electrocardiogram can be used as an auxiliary diagnostic index to evaluate pericardial thickness in tuberculous constrictive pericarditis.

## Introduction

Tuberculosis (TB) is one of the most prevalent infectious diseases in the world and a leading cause of infection-related death, particularly in developing countries [24]. Tuberculosis is the most common cause of constrictive pericarditis in low—and middle-income areas, with a reported incidence of 23–91% [25]. Tuberculous constrictive pericarditis is also known as “heart with shell” [21]. Tuberculous pericarditis has a high morbidity and mortality rate [17]. Tuberculous constrictive pericarditis is secondary to exudative pericarditis and is usually caused by Mycobacterium tuberculosis [9]. It is characterized by pericardial thickening, adhesion, fibrosis and calcification, which can impair diastolic function and lead to poor circulation. Surgical intervention is the most appropriate treatment for tuberculous constrictive pericarditis if the patient shows continuous clinical evidence after antituberculous therapy [23]. Pericardiectomy is the standard treatment for tuberculous constrictive pericarditis [18]. At present, chest radiography, computed tomography (CT) and echocardiography are the main diagnostic methods for tuberculous constrictive pericarditis [1, 19]. However, some patients without pericardial thickening and calcification also exhibit severe physiopathologic changes of constrictive pericarditis, suggesting that the diagnosis cannot be made solely on the basis of chest radiographs, computed tomography (CT) scans, and echocardiography. Therefore, supplementary examination and diagnostic indicators for the diagnosis of tuberculous constrictive pericarditis are of great significance to doctors' judgment of patients' condition, the implementation of pericardial dissection and the prognosis of patients.

Electrocardiogram (ECG) is the most common examination to record cardiac electrical activity produced by each cardiac cycle. ECG parameters, including heart rate, the time from the corrected ORS wave origin to T-wave terminal (QTc), ST-segment change and T-wave change, can be used to reflect cardiac dysfunctions, such as arrhythmia, cardiac hypertrophy and myocardial infarction (MI). In medicine, ECG parameters can be used as a predictor of many diseases. Some scholars have found that it’s neccessery for patients who have cardiovascular diseases, such as erectile dysfunction (ED) to pay attention to some ECG parameters such as Tp-e and Tp-e/QT in routine ECG evaluation [4]. And Tp-e interval and TP-e / QT measurements in the routine ECG evaluation of prediabetic patients are able to predict arrhythmia risk [4]. It can be predicted in HD patients for the risk of arrhythmia and sudden cardiac death by evaluating the electrocardiographic parameters showing ventricular repolarization and depolarization together [6]. In addition, Morris et al. first introduced the P-wave terminal force (PTF) in lead V1 (Ptfv1) measured by 12-lead ECG that can reflect the severity of the cardiac diseases. However, few studies have investigated the diagnostic value of ECG parameters in the diagnosis of tuberculous constrictive pericarditis.

In this study, we recruited patients with tuberculous constrictive pericarditis requiring pericardial dissection and performed descriptive statistics on their basic demographic characteristics, echocardiographic results and ECG parameters and other baseline characteristics. In addition, correlation analysis and nonparametric test were conducted between the values measured by echocardiography, chest CT and absolute value of Ptfv1 and those measured after pericardial dissection, respectively, to explore the auxiliary diagnostic value of Ptfv1 in the clinical diagnosis of tuberculous constrictive pericarditis.

## Methods

### Patients

From January 2018 to October 2022, after excluding 3 people who did not meet the inclusion criteria, there were 92 subjects in the final study, of which 63 were males (68.47%) and 29 were females (31.52%). According to the tuberculin test, T.pot -TB test, CT and echocardiography, and microbiology, the diagnosis was confirmed as tuberculous constrictive pericarditis. Microbiological confirmation here consists mainly of smear and culture confirmation of pericardial effusion. Patients meeting all of the following criteria were included in this study: (1) Patients with chronic systemic congestion and low cardiac output; (2) X-ray and CT examination showed obvious pericardial thickening or calcification; (3) The end diastolic difference between left and right ventricles was 5mmhg (1mmhg = 0.133 kPa); (4) Central venous pressure greater than 12 cmH2O; (5) The pathological diagnosis was granulomatous inflammation and caseous necrosis. Patients with rheumatic pericarditis, suppurative pericarditis, nonspecific pericarditis, coronary heart disease, pulmonary heart disease, and pericardial tamponade were excluded. Demographic and clinical characteristics of patients were recorded, including sex, age, comorbidities, and complications. Echocardiography was performed using a Philips iU 33 Doppler ultrasound instrument (Philips Healthcare, Andover, MA, USA). Philips 16-slice spiral CT was used to obtain chest CT data.

### Endotracheal intubation with general anesthesia pericardiectomy through median sternal incision

The patient was placed in a supine position with the chest protruding from the shoulder blade area of the back and splitting the median sternum. If there is retrosternal adhesion, separate the adhesion and use a thoracotomy to stretch the sternum on both sides. The pericardium is removed from the apex of the heart. In addition, pericardial adhesion is light, pericardial thickening is not obvious, and it is easy to peel. Cut the thickened pericardium with a blade one by one. There is often a loose connective tissue between the thickened pericardium and the outer membrane, in order to properly peel off the interface of the pericardium. After opening the thickened pericardium, the beating heart can be seen protruding outwards. After separating part of the pericardium, the assistant gently lifts the pericardium with pliers, and the surgeon gently presses the surface of the heart with his left hand to fully reveal the degree of adhesion between the thickened pericardium and the myocardium. If the adhesion is loose, it can be bluntly separated with finger gauze or peanut forceps, and the force part of separation should be on the pericardial surface. In case of cord or ribbon adhesion, sharp separation with scissors or surgical blades is required. If the adhesion is very tight, the original separation site should be abandoned, and the pericardium should be re-cut and separated at other sites, that is, easy first and difficult later. The scope of dissection was determined according to the patient's cardiac function and the degree of pericardial adhesion. The basic scope of the general stripping: should be the apex of the heart to be completely stripped; The left side is close to the left phrenic nerve; The fibrous constriction ring of atrioventricular groove and inferior vena cava entrance must be loosened. The sequence of dissection should be left ventricular → right ventricular outflow tract → atrioventricular coarctation ring → inferior vena cava ring band.

### ECG measurements

All patients received a standard 12-lead at rest ECG examination in supine position. The 12-lead ECG data were recorded by the trained ECGs doctors at double amplitude (20 mm/mV) and paper speed (50 mm/s) Ptfv1 was measured and calculated as following: (1) five continuous Ptfs in the sinus rhythm were selected and measured; (2) Ptfv1 value was recorded as 0 if P-wave showed in a vertical state; (3) the intersection of horizontal line derived from initial point of the P-wave and descending line of the P-wave was marked when P-wave presented in the positive and negative terminal deflection, then the vertical diameter and the horizontal spacing between the intersection and terminal point of the P-wave were recorded as the amplitude and duration of the negative terminal deflection of the P-wave, respectively; (4) mean amplitude (mm) and duration (s) were calculated based on five continuous amplitudes and durations of the negative terminal deflection of the P-wave; (5) product of the mean amplitude (mm) and duration (s) was recorded as Ptfv1 value (mm·s). The Ptfv1 abnormality was defined as Ptfv1 Value ≤ -0.04(mm·s) according to previously described.9 Additionally, the ECG parameters were recorded such as heart rate, and the presence or absence of bundle branch block or arrhythmia, which included atrial fibrillation, low voltage, ST-segment change, T-wave change and so on.

### Statistical analysis

SPSS 23.0 software (IBM Corporation, New York, USA) was applied to analyze all the data. Continuous variables were analyzed utilizing the normality test. Here, continuous variables with normal distribution were expressed as the mean ± standard deviation (SD). Classification variables were represented as percentage (%), and then analyzed by chi-square test. In the current study, *p* value < 0.05 were considered as statistically significant.

## Results

### Patient characteristics

The demographic and clinical characteristics of patients with tuberculous constrictive pericarditis are shown in Table 1. Ninety-five patients with tuberculous constrictive pericarditis who needed pericarditis dissection in a hospital from January 2018 to October 2022 were collected, and 3 patients who did not meet the criteria were excluded, a total of 92 cases. The mean age of subjects was 57.59 years (SD = 15.48). There were 63 males (68.48%) and 29 females (31.52%). There were 18 cases of hypertension (19.57%), 9 cases of diabetes (9.78%), 2 cases of coronary artery disease (2.17%), and 0 cases of cardiomyopathy and congenital heart disease.

**Table 1:** The clinical and demographic characteristics of patients with tuberculous constrictive pericarditis

	Me (SD)	N (%)
Age	57.59 (15.48)	
Sex		
Male		63 (71.74)
Female		29 (28.26)
Comorbidity and complication		
Hypertension		18 (19.57)
Diabetes		9 (9.78)
Coronary artery disease		2 (2.17)
Cardiomyopathy		0 (0.00)
Congenital heart disease		0 (0.00)

ECG parameters and other measurements were showed in Table 2. The average heart rate of the subjects was approximately 102 beats per minute (SD = 12.69). In addition, ECG results showed that there were 54 patients (58.70), 85 patients (92.39), 53 patients (57.61), 15 patients (16.30), 10 patients (10.87) and 2 patients (2.17) with ST segment changes, T wave changes, limb conduction low voltage, chest conduction low voltage, arrhythmia and bundle branch block, respectively. Among 92 subjects, the mean left atrial diameter was 34.82, the mean left ventricular end-diastolic diameter was 41.71, and the mean left ventricular ejection fraction was 59.58. The mean measured value of Ptfv1 was 0.04 (SD = 0.01). In addition, the mean echocardiographic measurements were 4.67 (SD = 4.05), the mean chest CT measurements were 16.54 (SD = 6.80), and the mean postoperative measurements were 8.92 (SD = 3.63).

**Table 2:** Electrocardiograph parameters and other measurements of patients with tuberculous constrictive pericarditis

	Me (SD)	N (%)
ST segment Abnormalities		54 (58.70)
T-wave Abnormalities		85 (92.39)
Limb conduction low voltage		53 (57.61)
Chest conduction low voltage		15 (16.30)
Arrhythmia		10 (10.87)
Bundle branch block		2 (2.17)
Ptfv1 value	0.04 ± 0.01	
Echocardiogram measurements	4.67 ± 4.05	
Chest CT measurements	16.54 ± 6.80	
Values measured after pericardial dissection	8.92 ± 3.63	
Left atrial diameter	34.82 ± 5.41	
Left ventricular end-diastolic diameter	41.71 ± 7.57	
Left ventricular ejection fraction	59.58 ± 8.00	
Heart rate	101.98 ± 12.69	

### Correlation analysis

**Fig. 1:**
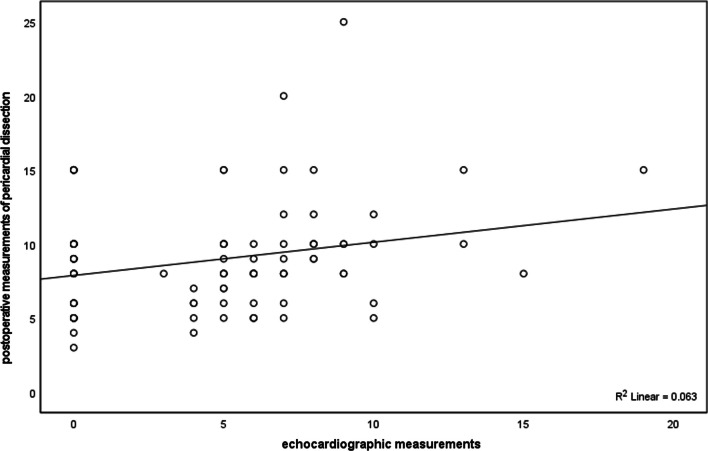
Scatter plots between echocardiographic measurements and postoperative measurements of pericardial dissection

**Fig. 2:**
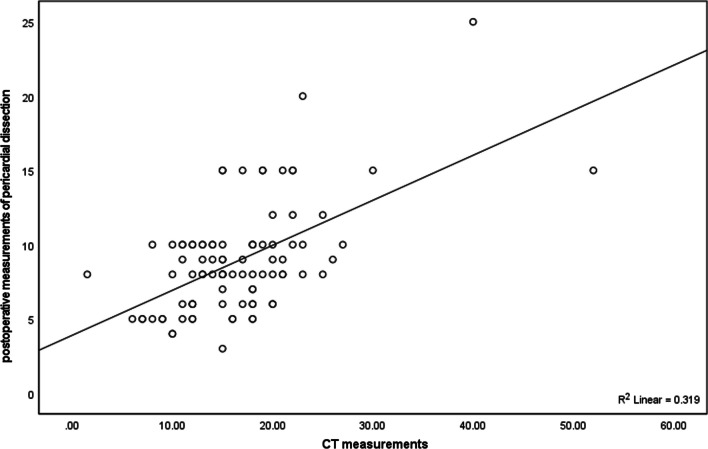
Scatter plots between chest CT measurements and postoperative measurements of pericardial dissection

The value of echocardiography, chest CT and absolute value of Ptfv1 of 92 subjects were respectively compared with the value of postoperative pericardial dissection by Pearson correlation analysis. The results of this study showed (Table 3) that the correlation coefficient between the postoperative measurements of pericardial dissection and those of echocardiography was 0.250, which was a positive correlation, and its significance was *p* = 0.016 < 0.05, so the correlation coefficient between the two is significantly non-zero. Similarly, there is a strong correlation between the measured value after pericardial dissection and the measured value of chest CT, with a correlation coefficient of 0.565, *p* = 0.000 < 0.05, and the correlation coefficient between postoperative measurements and the absolute value of Ptfv1 was 0.095, and *p* = 0.370 > 0.05, so the correlation between the two was weak, that is to say, the greater the absolute value of Ptfv1, the greater the postoperative measurement value of pericardial dissection is basically non-existent. In addition, scatter plots were made of echocardiographic measurements, chest CT measurements, and postoperative measurements of pericardial exfoliation respectively (Figs. 1 and 2).

**Table 3:** the correlation analysis between echocardiographic measurements, chest CT measurements, absolute value of Ptfv1 and postoperative measurements

	Pearson	*p*	N
Echocardiographic measurements	0.250^**^	0.016	92
Chest CT measurements	0.565^***^	< 0.001	91
Absolute value of Ptfv1	0.095*	0.082	92

*p < 0.1, **p < 0.05, ***p < 0.001

**Fig. 3:**
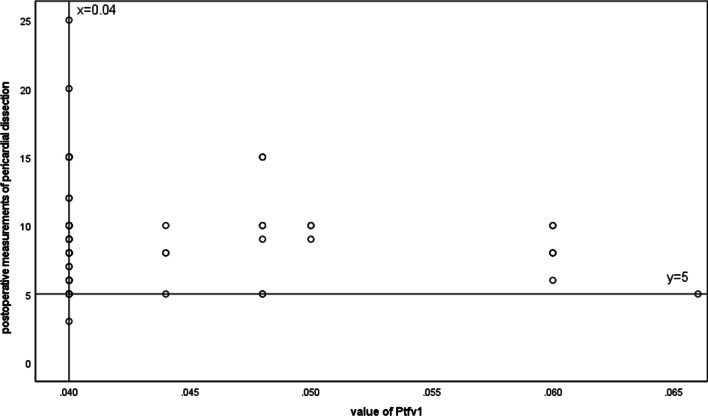
Scatter plots between value of Ptfv1 and postoperative measurements of pericardial dissection

In addition, R was used to make between Ptfv1 and postoperative measurements (Fig. 3). y = 5 is expressed as postoperative measurements = 5, x = 0.04 is expressed as absolute value of Ptfv1 = 0.04. It can be shown that the vast majority of postoperative measurements are ≥ 5 in the absolute value of Ptfv1 ≥ 0.04.

### Non-parametric test

Based on the above, we proposed the following hypotheses: H0: absolute value of Ptfv1 ≥ 0.04 after middle stripping ≥ 5 and absolute value of Ptfv1 ≥ 0.04 after middle stripping; H1: absolute value of Ptfv1 ≥ 0.04 middle exfoliation postoperative value ≥ 5 and Ptfv1 absolute value ≥ 0.04 middle exfoliation postoperative value; The distribution of 5 is not 0.95:0.05. The non-parametric Chi-square test is used to verify the validity of the conjecture. Chi-square test results show that chi-square = 2.074, p = 0.15, the null hypothesis cannot be rejected. The distribution of the value of Ptfv1 ≥ 5 was higher than the value of Ptfv1 < 5 after pericardiectomy (0.95:0.05) in the absolute value of Ptfv1 ≥ 0.04 which measured before pericardiectomy.

## Discussion

In the present study, our results show that when electrocardiogram Ptfv1 absolute value ≥ 0.04 in patients with tuberculous constrictive pericarditis, the pericardial thickness may be >  = 0.5 cm. The absolute value of Ptfv1 in ECG can be used as an auxiliary diagnostic index to evaluate pericardial thickness in tuberculous constrictive pericarditis.

Ptfv1 had been regarded as a specific indicator to reflect left atrial structural abnormalities, and was related to the diastolic function of left ventricular [5, 14]. In an early study, normal reference value of Ptfv1 is defined as 0.03 (mm·s), and about 7% of abnormal Ptfv1 is apparently appeared in healthy middle-aged men [8]. Recently, Ptfv1 ≤ -0.04 (mm·s) is considered as abnormal Ptfv1 [7].Consequently, the present study selected -0.04 (mm·s) as thereference value of Ptfv1. Generally, the abnormality of Ptfv1 could be explained as the following two mechanisms: (1) the left atrial time depolarization and left atrial depolarization vector would be increased when left atrial load increase, hypertrophy, ischemia and fibrosis; (2) right atrial load increase and hypertrophy as well as prolonged interatrial bundle conduction interval could induce increased amplitudes and broadened durations of the negative terminal deflection of the P-wave [12, 13, 15, 16]. Previous studies indicated that abnormal Ptfv1 was associated with left ventricular diastolic dysfunction, atrial fibrillation, stroke, congestive heart failure and mortality [2, 3, 10, 11, 20]. Abnormal Ptfv1 could also predict the risk of adverse cardiovascular events.In addition, Ptfv1 was proved to be related to the pulmonary emphysema.

These results all indicate that Ptfv1 has clinical diagnostic value in some diseases. However, few studies have been conducted on the diagnostic value of Ptfv1 in tuberculous constrictive pericarditis. This study found that Ptfv1 has a certain auxiliary diagnostic value for tuberculous constrictive pericarditis, which may be due to pericardial thickening, adhesion, fibrosis and calcification of patients leading to poor blood circulation, thus increasing the left and right atrial load, and eventually leading to abnormal Ptfv1 [22]. Abnormal Ptfv1 is closely associated with tuberculous constrictive pericarditis. Abnormal Ptfv1 by electrocardiogram can be used as an important auxiliary diagnostic indicator in the clinical diagnosis of tuberculous constrictive pericarditis, and can make up for the limitations of X-ray, CT and echocardiography in the diagnosis of tuberculous constrictive pericarditis. Abnormal Ptfv1 has important diagnostic value in the clinical diagnosis of tuberculous constrictive pericarditis.

In summary, our findings suggest that abnormal Ptfv1 is associated with tuberculous constrictive pericarditis, and Ptfv1 is a potential auxiliary diagnostic indicator for the diagnosis of tuberculous constrictive pericarditis.

## Strengths and limitations

There are two limitations to this study. Firstly, this is a retrospective study with a small sample size, and the results of this study need to be confirmed by more prospective studies with a large sample size. Secondly, in order to confirm that Ptfv1 is an auxiliary diagnostic criterion for the diagnosis of tuberculous constrictive pericarditis, this study needs to set a reasonable control group for further exploration. In addition, this issue will also be investigated in the future.

## Conclusion

In conclusion, this study reports that the absolute value of Ptfv1 in ECG can be used as an auxiliary diagnostic index to evaluate pericardial thickness in tuberculous constrictive pericarditis. This study empirically verified that abnormal Ptfv1 is closely associated with tuberculous constrictive pericarditis. Abnormal Ptfv1 by electrocardiogram can make up for the limitations of X-ray, CT and echocardiography in the diagnosis of tuberculous constrictive pericarditis. Abnormal Ptfv1 has important diagnostic value in the clinical diagnosis of tuberculous constrictive pericarditis.

## Data Availability

The datasets generated and analyzed during the current study are available from the corresponding author on reasonable request.
